# Quality of life in patients with hypoparathyroidism receiving standard treatment: an updated systematic review

**DOI:** 10.1007/s12020-024-03807-2

**Published:** 2024-04-05

**Authors:** Matthias Büttner, Susanne Singer, Katherine Taylor

**Affiliations:** 1 Institute of Medical Biostatistics, Epidemiology and Informatics (IMBEI), University Medical Center Mainz, Mainz, Germany; 2 University Cancer Centre, Mainz, Germany

**Keywords:** Hypoparathyroidism, Quality of life, Well-being, Systematic review

## Abstract

**Purpose:**

Hypoparathyroidism is defined by hypocalcemia with inappropriately normal or low parathyroid hormone levels. The current standard treatment consists of lifelong calcium and/ or vitamin D supplementation. Even while on stable treatment regimens, hypoparathyroid patients might still suffer from symptoms that can negatively impact their quality of life.

**Methods:**

A systematic literature review to identify the current knowledge regarding quality of life in patients with hypoparathyroidism receiving standard treatment was performed on November 1st, 2023. PubMed as well as Web of Science were searched. The systematic review is registered in PROSPERO (#CRD42023470924).

**Results:**

After removal of duplicates, 398 studies remained for title and abstract screening, after which 30 were included for full-text screening. After exclusion of seven studies with five studies lacking a control population, one using a non-validated questionnaire, and one being a subsample of the larger included study, 23 studies were included in this systematic review. The majority of the included studies used a guideline-conform definition of hypoparathyroidism, and the SF-36 was the most often applied tool. Almost all studies (87%) reported statistically significantly lower scores in at least one quality of life domain compared to a norm population or controls.

**Conclusion:**

Patients with hypoparathyroidism receiving standard treatment report impairments in quality of life. The reasons for these impairments are probably multifaceted, making regular monitoring and the inclusion of various professionals necessary.

## Introduction

Hypoparathyroidism (hypoPT) is a rare endocrine disorder defined by hypocalcaemia with inappropriately normal or low parathyroid hormone (PTH) levels [1–3]. For approximately 75% of cases, thyroid or parathyroid surgery is the main cause for hypoPT [4]. Other causes of hypoPT can be autoimmune disease or genetic variations, but the causes for a considerable proportion of the non-surgical cases remain unclear and are considered as idiopathic [5, 6]. The prevalence of hypoPT lies between 5.3–40.0 per 100,000 individuals, with differences between countries [5, 7–12]. The standard treatment consists of lifelong calcium and/or vitamin D supplementation with regular monitoring [13], but this treatment does not restore the regular calcium/phosphorus homeostasis [2, 10]. Patients with hypoPT suffer from a variety of symptoms such as tingling, cramps, and gastrointestinal problems even long after diagnosis. These symptoms can have an impact on hypoPT patients’ quality of life (QoL) [10, 14, 15]. The importance of QoL is also highlighted in the European [13] und North-American [16] guidelines for the management of hypoPT, stating that treatment should be personalized and focused on overall well-being and QoL. Especially with new treatments emerging within the last years, the focus on QoL is becoming more important [17–21].

To better understand the relationship of hypoPT with QoL, we had conducted a systematic review in 2016 [22]. Since that time, new studies have been published, warranting an update of this overview.

The aim of this updated systematic review is to give an overview of hypoPT patients’ QoL receiving standard treatment and to provide information on the definition of hypoPT and questionnaires used in the respective studies.

## Methods

### Search strategy

On November 1st, 2023, a systematic literature search in PubMed and Web of science was performed. This systematic review was performed in accordance with the PRISMA statement [23]. The search terms were the same as in the previous systematic review, namely “hypoparathyroidism” or “hypocalcemia” or “hypocalcaemia” in combination with the terms “quality of life” or “qol” or “well-being” within the title or abstract. Grey literature and references of all selected publications were checked. No further restrictions were applied. The systematic review is registered in PROSPERO (#CRD42023470924).

### Eligibility criteria

The following inclusion criteria were applied (a) adult patients with a diagnosis of hypoPT for more than 6 months, (b) QoL had to be measured with a validated instrument, (c) patients needed to be on current conventional treatment consisting of calcium and/or vitamin d, (d) QoL outcome measure needed to be compared to a reference population or controls. Exclusion criteria were: (a) pediatric patients, (b) patients with untreated hypoPT, (c) case reports, (d) and review papers.

### Manuscript screening and data abstraction

All titles and abstracts were screened by two independent reviewers (MB and KT) using Covidence [24]. Full texts of all eligible studies were acquired and evaluated by the two independent reviewers. If disagreement regarding inclusion of a study occurred, this was solved by discussion between the two reviewers resulting in a consensual decision. Extracted data was stored in an electronic file including: authors, year of publication, journal name, study design, sample population, reference population, and QOL instruments used. For intervention studies investigating new treatments in patients with hypoPT, QoL at baseline was extracted for the impact of standard treatment on QoL. When available, QoL scores were extracted as well. QoL scores available only in figures were not extracted.

### Quality assessment

Quality assessment of the studies was carried out by using the Newcastle-Ottawa Quality assessment scale for cross-sectional studies as proposed by Herzog et al. [25]. Using this assessment tool, studies can score a total of ten stars, indicating the highest quality. The scoring can be subdivided into three domains consisting of *selection* (maximum five stars), *comparability* (maximum two stars), and *outcome* (maximum three stars) [25]. Seven studies were not of cross-sectional origin but were considered cross-sectional as only information on baseline comparisons was extracted for this systematic review.

## Results

### Study selection

**Fig. 1:**
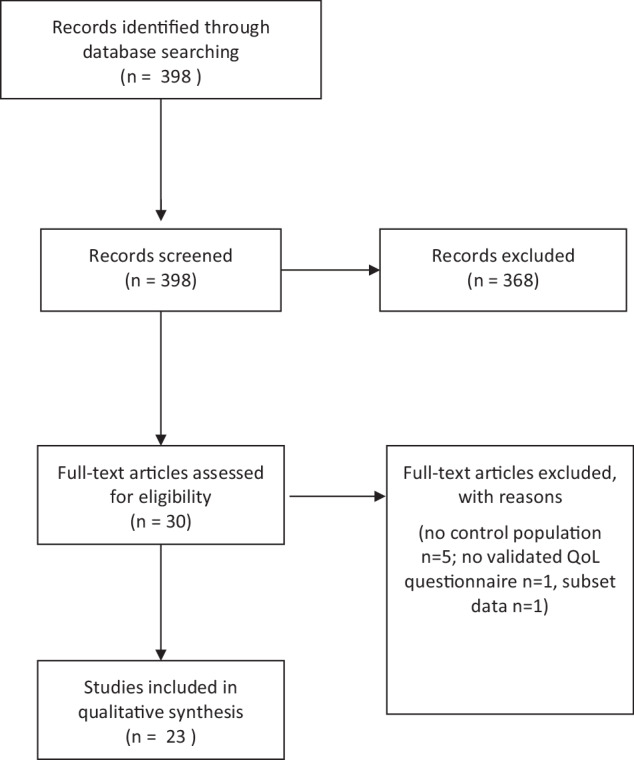
Flow chart of the literature selection process

The literature search resulted in 398 papers (after removal of 836 duplicates). After title and abstract screening, 30 studies remained for full-text review. Of these, five were removed because they lacked a control population, one was removed because QoL was assessed using a non-validated questionnaire and one was removed because its data was used in a larger study included in this analysis, leaving 23 studies eligible for data extraction. The full literature screening process is presented in Fig. 1. The quality assessments of all included studies can be found in Table 1; no studies were excluded based on their quality. Regarding the control population, 11 studies [5, 14, 17, 19, 20, 26–31] used norm population data for comparison while 12 studies used controls [32–43]. Information on the hypoPT group and comparison group for each study can be found in Table 2.

**Table 1:** Quality assessment of the included studies

Author	Selection				Comparability		Outcome		Total
	Representativeness	Sample size	Non-respondents	Exposure	Most important factors	Additional factors	Assessment	Statistical test	
Arlt et al. [32]		*		**	*	*	**	*	***** ***
Arneiro et al. [33]	*	*		**	*	*	**	*	***** ****
Astor et al. [5]	*	*	*	**	*	*	**	*	***** *****
Büttner et al. [34]		*		**	*	*	**	*	***** ***
Büttner et al. [14]	*	*		*	*	*	**	*	***** ****
Cherchir et al. [35]	*	*		**	*	*	**	*	***** ****
Cusano et al. [26]	*	*		**	*	*	**	*	***** ****
Frey et al. [36]		*		**	*	*	**	*	***** ***
Hepsen et al. [37]	*	*		**	*	*	**	*	***** ****
Hillary et al. [38]		*		*	*	*	**	*	***** **
Jorgensen et al. [39]		*	*	**	*	*	**	*	***** ****
Khan et al. [27]	*	*		**			**		***** *
Khan et al. [17]	*	*		**			**		***** *
Kontogeorgos et al. [40]	*	*		**	*	*	**	*	***** ****
Mazoni et al. [41]		*		**	*	*	**	*	***** ***
Roszko et al. [28]	*	*		**	*	*	**	*	***** ****
Sikjaer et al. [29]	*	*		**	*	*	**	*	***** ****
Sikjaer et al. [42]		*		**	*	*	**	*	***** ***
Song et al. [43]		*		**	*	*	**	*	***** ***
Tabacco et al. [19]	*	*		**	*	*	**	*	***** ****
Underbjerg et al. [30]		*		**	*	*	**	*	***** ***
Vokes et al. [20]	*	*		**			**	*	***** **
Wilde et al. [31]	*	*		*			**	*	***** *

Star (*) represents whether a study fulfills the respective criteria. In total are the sum of all stars

**Table 2:** Overview of the included studies

Author	hypoPT population	Norm or control population	Definition of hypoPT	QoL insturments
Arlt et al. [32]	25 women with postsurgical hypoPT	25 women with a history of thyroid surgery but normal parathyroid function	Guideline definition	SCL-90-R; B-L Zerssen; GBB-24
Arneiro et al. [33]	37 postsurgical or autoimmune hypoPT patients	20 patients who had undergone total thyroidectomy	Guideline definition	SCL-90-R
Astor et al. [5]	283 hypoPT patients	Norwegian normative data	Guideline definition	SF-36
Büttner et al. [34]	17 hypo patients at least nine months after treatment for TC	72 patients without hypoPT at least nine months after treatment for TC	Confirmed diagnosis with information on diagnostic criteria	EORTC QLQ-C30
Büttner et al. [14]	264 hypoPT patients	Age- and sex matched German norm population	Self-reported by the patient	EORTC QLQ-C30
Cherchir et al. [35]	53 hypoPT patients	53 controls matched for surgical status, age, sex, bodymass index, and socioeconomic conditions	Guideline definition	SF-36
Cusano et al. [26]	69 hypoPT patients	US norm population	Guideline definition	SF-36
Frey et al. [36]	45 postsurgical hypoPT patients	96 controls without hypoPT after total thyroidectomy	Guideline definition	SF-36
Hepsen et al. [37]	160 hypoPT patients	148 age- and sex matched controls	Guideline definition	SF-36
Hillary et al. [38]	89 postsurgical hypoPT patients	350 controls without hypoPT after thyroid surgery	Self-reported by the patient and guideline definition	SF-36; hcSS
Jorgensen et al. [39]	14 postsurgical hypoPT patients	28 patients without hypoPT after total thyroidectomy	Guideline definition	SF-36
Khan et al. [27]	44 hypoPT patients	US norm population	Guideline definition	SF-36; HPES
Khan et al. [17]	61 hypoPT patients	US norm population	Guideline definition	SF-36; HPES
Kontogeorgos et al. [40]	203 hypoPT patients	414 controls from a population based cohort study	Guideline definition	SF-36
Mazoni et al. [41]	89 hypoPT patients after treatment for differentiated TC	89 age- and sex matched controls without hypoPT after treatment for differentiated TC	Guideline definition	SF-36; WHO-5
Roszko et al. [28]	31 hypoPT patients	US norm population	Guideline definition	SF-36
Sikjaer et al. [29]	62 hypoPT patients	US norm population	Guideline definition	SF-36; WHO-5
Sikjaer et al. [42]	22 postsurgical hypoPT patients	22 postsurgical patients without hypoPT22 healthy controls	Guideline definition	SF-36; WHO-5
Song et al. [43]	101 non-surgical hypoPT patients	101 healthy controls	Guideline definition	SF-36
Tabacco et al. [19]	20 hypoPT patients	US norm population	Guideline definition	SF-36
Underbjerg et al. [30]	57 non-surgical hypoPT patients	US norm population	Confirmed diagnosis with information on diagnostic criteria	SF-36; WHO-5
Vokes et al. [20]	122 hypoPT patients	US norm based population	Guideline definition	SF-36
Wilde et al. [31]	60 hypoPT patients	Norm population data	Self-reported by the patient	SCL-90-R; B-L Zerssen; GBB-24

### Diagnosis of hypoPT

In most studies (18 of 23) [5, 17, 19, 20, 26–29, 32, 33, 35–37, 39–43], the hypoPT diagnosis was confirmed by laboratory parameters, namely low or inappropriately low PTH levels in combination with hypocalcemia or low calcium levels for at least six months. Two studies [30, 34] reported that hypoPT was confirmed by the treating physician without further information on the diagnostic criteria, and two studies [14, 31] relied on a self-reported diagnosis from the patient. Hillary et al. [38] used a combined study population for their analysis, including patients with a confirmed diagnosis of low PTH in combination with hypocalcemia or low calcium levels as well as patients with a self-reported diagnosis enrolled from self-help organizations.

### Quality of life assessment tools

The short-form, 36-item generic Medical Outcomes Survey (SF-36) [44] was used by the majority (19 of 23) of the studies [5, 17, 19, 20, 26–31, 35–43] (Table 2). The 36 items in the SF-36 cover eight domains (*physical functioning*, *role functioning related to physical health*, *bodily pain*, *general health*, *vitality*, *social functioning*, *role functioning related to emotional problems*, *mental health*), as well as a physical component score (PCS) and mental health component score (MCS). Other generic questionnaires such as the 5-item World Health Organization Well-Being Index (WHO-5) [45] (used four times) [29, 30, 41, 42], the revised version of the Symptom Checklist 90 (SCL-90-R) [46] (used three times) [31–33], the Complaint List of von Zerssen (B-L Zerssen) [47] (used two times) [31, 32], and the short form of the Gießen Complaint List (GBB-24) [48] (used two times) [31, 32] were also applied. The European Organization for Research and Treatment of Cancer’s quality of life core questionnaire (EORTC QLQ-C30) [49] was used two times [14, 34]. Four studies used a hypoparathyroidism-specific instrument: the Hypoparathyroid Patient Experience Scale (HPES) [50] (used two times) [17, 27], the Hypoparathyroid Patient Questionnaire (HPQ-28) [31] (used one time) [14], and the hypocalcaemia symptom score (HcSS) (used one time) [38].

### Quality of life compared to norm populations

Of the eleven studies comparing hypoPT patients’ QoL to norm populations, eight studies [5, 14, 19, 26, 28–31] found at least one QoL-related domain where hypoPT patients reported statistically significantly lower scores compared to the norm population. Vokes et al. [20] reported lower SF-36 baseline scores for the hypoPT patients compared to the norm population but the differences were small. In the two studies by Khan et al. [17, 27], the PCS of the SF-36 was lower compared to the norm population without any reporting of statistical tests. Three studies [19, 26, 30] reported statistically significantly lower SF-36 scores across all domains compared to a norm population. Two of those studies [19, 26] had mixed hypoPT populations (post-surgical and non-surgical hypoPT), and the study of Underbjerg et al. [30] only looked at non-surgical hypoPT. In Astor et al. [5] patients with hypoPT had statistically significantly lower scores in *physical functioning* (hypoPT: 74.2; norm: 87.2) *role functioning related to physical health* (hypoPT: 44.9; norm: 77.9), *bodily pain* (hypoPT: 58.1; norm: 75.1), *general health* (hypoPT: 50.7; norm: 76.8), *vitality* (hypoPT: 42.2; norm: 60.0), *role functioning related to emotional problems* (hypoPT: 65.1; norm: 81.6), *mental health* (hypoPT: 70.5; norm: 78.8) and *social functioning* (hypoPT: 68.5; norm: 85.5, all *p* < 0.05), compared to a Norwegian norm population. Compared to patients with nonsurgical hypoPT, patients with postsurgical hypoPT scored lower in *role functioning related to physical health* (postsurgical: 39.2; nonsurgical: 58.6; *p* = 0.002), *bodily pain* (postsurgical: 55.3; nonsurgical: 63.8; *p* = 0.03), and *vitality* (postsurgical: 40.0; nonsurgical: 46.4; *p* = 0.04) [5]. Rozsko et al. [28] found lower scores in *role functioning related to physical health*, *general health*, *vitality*, and *social functioning*, while Sikjaer et al. [29] reported lower scores for *physical functioning* (45.3 ± 9.2), *role functioning related to physical health* (42.6 ± 11.7), *bodily pain* (43.8 ± 10.9), *general health* (42.5 ± 10.6), *vitality* (43.9 ± 11.9), *role functioning related to emotional problems* (45.3 ± 11.2), and *social functioning* (47.3 ± 9.9, all *p* < 0.05), both using the SF-36 with its norm data (mean: 50.0; SD:10). Using the functioning scales of the EORTC QLQ-C30, Büttner et al. [14] found lower scores across all five domains (*physical functioning* (hypoPT: 74.0; norm: 82.0; *p* < 0.01), *role functioning* (hypoPT: 63.6; norm: 80.3; *p* < 0.01), *social functioning* (hypoPT: 61.7; norm: 85.1; *p* < 0.01), *cognitive functioning* (hypoPT: 56.9; norm: 85.4; *p* < 0.01), and *emotional functioning* (hypoPT: 46.9; norm: 75.1; *p* < 0.01)) compared to an age and sex-adjusted norm population. Compared to a norm population, Wilde et al. [31] reported more symptoms for *somatization* (hypoPT: 1.32; norm: 0.47; *p* < 0.001), *obsessive-compulsive symptoms* (hypoPT: 0.91; norm: 0.45; *p* < 0.001), *depression* (hypoPT: 0.92; norm: 0.44; *p* < 0.001), and *anxiety* (hypoPT: 0.82; norm: 0.34; *p* < 0.001) with the SCL-90-R. Additionally, patients scored higher (more complaints) in four (*exhaustion* (hypoPT: 11.17; norm: 5.55; *p* < 0.001), *heart complaints* (hypoPT: 6.07; norm: 3.41; *p* = 0.009), *pain in the limbs* (hypoPT: 11.36; norm: 6.51; *p* < 0.001), and *global score of discomfort* (hypoPT: 34.17; norm: 18.18; *p* < 0.001)) of the five GBB-24 domains.

### Quality of life compared to controls

Four [35, 37, 40, 43] of the 12 studies used clinical controls as comparison groups. Compared to age- and sex matched controls, hypoPT patients in three studies [35, 37, 43] had statistically lower QoL scores in all eight domains of the SF-36. Song et al. [43] report that patients with hypoPT had statistically significantly lower QoL scores in all eight domains compared to age- and sex matched controls. In Hepsen et al. [37] median QoL scores were statistically significantly lower in hypoPT patients compared to age- and sex matched controls (*physical functioning* (hypoPT: 70; controls: 95; *p* < 0.001) *role functioning related to physical health* (hypoPT: 50; controls: 100; *p* < 0.001), *bodily pain* (hypoPT: 57.5; controls: 90; *p* = 0.001), *general health* (hypoPT: 42.5; controls: 70; *p* = 0.001), *vitality* (hypoPT: 40; controls: 60; *p* = 0.001), *role functioning related to emotional problems* (hypoPT: 33.3; controls: 66.7; *p* = 0.002), *mental health* (hypoPT: 60; controls: 72; *p* = 0.001) and *social functioning* (hypoPT: 62.6; controls: 7; *P* = 0.001) with postsurgical hypoPT patients having lower scores in *vitality* (postsurgical: 40; nonsurgical: 50; p = 0.003), and *mental health* (postsurgical: 60; nonsurgical: 76; p = 0.001) compared to nonsurgical hypoPT patients. Cherchir et al. [35] also reported statistically significantly lower scores in all eight SF-36 domains (*physical functioning* (hypoPT: 75; controls: 88.6; *p* < 0.001) *role functioning related to physical health* (hypoPT: 34.4; controls: 67.9; *p* < 0.001), *bodily pain* (hypoPT: 47.4; controls: 71.9; *p* < 0.001), *general health* (hypoPT: 25.8; controls: 48.5; *p* < 0.001), *vitality* (hypoPT: 29.3; controls: 52.3; *p* < 0.001), *role functioning related to emotional problems* (hypoPT: 22.5; controls: 66.5; *p* < 0.001), *mental health* (hypoPT: 41.6; controls: 62.5; *p* < 0.001) and *social functioning* (hypoPT: 40.7; controls: 70.3; *p* < 0.001) when comparing hypoPT patients to age- and sex matched controls. Kontogeorgos et al. [40] reported statistically lower PCS (hypoPT: 40.0; controls: 51.2; *p* < 0.001) and MCS (hypoPT: 43.1; controls: 56.1; *p* < 0.001) compared to age- and sex matched controls for their 203 hypoPT patients. For their mixed hypoPT population, Arneiro et al. [33] included 20 patients with post-surgical hypoPT and 17 with autoimmune hypoPT. As a control group 20 patients without hypoPT after total thyroidectomy were used. HypoPT patients had greater reported higher symptom burden by greater Global Severity Index score (hypoPT: 1.1; controls: 0.8; *p* = 0.03) of the SCL-90R. The remaining seven studies [32, 34, 36, 38, 39, 41, 42] focused on patients with post-surgical hypoPT and used patients after thyroidectomy without hypoPT as controls. Hillary et al. [38] enrolled 89 postsurgical hypoPT patients and 350 patients without hypoPT after surgery for thyroid disease or primary hyperparathyroidism as controls and reported statistically significantly differences only for *vitality* using the SF-36. Frey et al. [36] and Jorgensen et al. [39] compared postsurgical hypoPT patients with controls who also received total thyroidectomy for various reasons but did not develop hypoPT using the SF-36. Compared to the controls the hypoPT patients reported a statistically significantly lower median MCS ratio (hypoPT: 0.88; controls: 1.04; *p* = 0.003) [36] and lower scores in six (*physical functioning* (hypoPT: 66.7; controls: 81.9; *p* = 0.03), *role functioning related to physical health* (hypoPT: 48.2; controls: 80.8; *p* = 0.02), *bodily pain* (hypoPT: 54.9; controls: 77.3; *p* = 0.04), *general health* (hypoPT: 33.2; controls: 46.9; *p* < 0.01), *vitality* (hypoPT: 31.7; controls: 58.2; *p* < 0.01), and *mental health* (hypoPT: 63.1; controls: 77.3; *p* = 0.02)) of the eight SF-36 domains [39]. Using the SCL-90R, GBB-24, and B-L Zerssen, Arlt et al. [32] showed that 25 women with postsurgical hypoPT compared to 25 women without hypoPT matched for age and time since surgery reported significantly higher global complaint scores for the three questionnaires used. Two studies [34, 41] solely included patients with and without hypoPT after treatment for thyroid cancer. Büttner et al. [34] analyzed data from the development of thyroid cancer-specific EORTC thyroid module (EORTC QLQ-THY34) [51]. Seventeen patients with hypoPT at least nine months after treatment for thyroid cancer were compared to 72 controls without hypoPT at least 9 months after treatment for thyroid cancer using the EORTC QLQ-C30. HypoPT patients had statistically significantly lower scores compared to the controls in five (*global quality of life* (hypoPT: 51.0; controls: 68.5; *p* = 0.03), *physical functioning* (hypoPT: 66.7; controls: 82.7; *p* = 0.01), *role functioning* (hypoPT: 66.7; controls: 82.7; *p* = 0.02), *emotional functioning* (hypoPT: 56.9; controls: 80.0; *p* < 0.01), and *social functioning* (hypoPT: 69.6; controls: 86.0; *p* = 0.04),) of the six functioning domains [34]. In Mazoni et al. [41], 89 hypoPT patients after treatment for differentiated thyroid cancer were compared to 89 controls without hypoPT using the SF-36, resulting in statistically significantly lower scores in *physical functioning*, *general health*, *role functioning related to emotional problems*, PCS and MCS for the hypoPT patients. The study be Sikjaer et al. [42] is the only one that used more than one control population: 22 postsurgical hypoPT patients with well-substituted hypothyroidism were compare to 22 postsurgical patients with well-substituted hypothyroidism but without hypoPT and with 22 controls. Groups were matched for age, gender and time since surgery (not for the healthy controls). Compared to the postsurgical controls, the hypoPT patients reported statistically significantly lower scores in the *physical health* and *role functioning related to physical health* domains of the SF-36, while compared to the healthy controls they scored statistically significantly lower in seven (all except *role functioning related to emotional problems*) of the eight SF-36 domains.

## Discussion

This updated systematic review summarizes more evidence for the association of QoL with hypoPT in patients receiving standard treatment and provides an overview regarding the definition of hypoPT used in the studies, the QoL instruments used.

The majority of studies included in this systematic review used a definition of hypoPT as it is stated in the guidelines, namely low calcium and an inappropriately low PTH for at least 6 months [13, 16]. An accurate and comprehensible diagnosis of hypoPT is crucial in order to provide the best treatment to the patient. Two systematic reviews [52, 53] have investigated different definitions of hypoPT used in studies. In 2010, Mehanna et al. [53] conducted a systematic review in order to identify different definitions of hypoPT used in the literature and to apply them to their cohort of 202 patients undergoing total or hemithyroidectomy. The ten main different definitions identified in this review resulted in a cumulative incidence of post-surgical hypoPT ranging from 0 to 46% showing the high heterogeneity among the different definitions. Harslof et al. [52] conducted a systemic review in 2019 looking at 89 studies and their definition of hypoPT. They identified 20 different definitions and 16 studies not reporting any information on the definition of hypoPT. Four of the studies in our systematic review did not provide a clear definition of hypoPT, but two studies enrolled their participants from hypoPT self-help organizations with extensive questions regarding their diagnosis of hypoPT within the used surveys. Therefore, we are confident that only patients with a clear diagnosis of hypoPT were included. The two other studies did not report a clear definition of hypoPT, but patients were diagnosed in a hospital setting by their treating physician ensuring an appropriate hypoPT diagnosis.

Only four studies (17%) in our systematic review used hypoPT-specific QoL instruments while the rest used generic or cancer-specific instruments. The problem with generic QoL instruments is that they might miss symptoms or QoL impairments of the specific disease [54, 55]. Even though the majority of studies reported impairments in QoL in hypoPT, some did not or only for very few domains [17, 20, 27], leaving the question open as to whether a disease-specific tool might have found more or different impairments or improvements. This may well be the case, as studies have identified hypoPT symptom burden to be associated with impairments in QoL [41, 56]. Because the hypoPT-specific questionnaires only have been recently developed [31, 50], the included studies cannot be criticized for not applying them. For future studies, it is important to use a tool which covers generic QoL domains as well as the hypoPT-specific symptoms or domains.

HypoPT patients receiving standard treatment report impairments in QoL compared to norm populations or matched controls. HypoPT patients report QoL scores comparable to patients with chronic heart disease or diabetes [57], or worse compared to patients with other chronic diseases such as Addison’s disease or congenital adrenal hyperplasia [58, 59]. There are various possible explanations for the impairments in QoL. The lack of PTH itself might negatively impact QoL as PTH receptors have been found in muscle cells, the central nervous system, and several brain regions [60–64]. Song et al. [43] found a potential association of current PTH levels and QoL, but this finding was not confirmed by Hepsen et al. [37]. Studies investigating new treatments using synthetic PTH have shown promising results also in relation to QoL [17, 18, 21, 29]. Another potential factor influencing QoL in hypoPT patients is the occurrence and severity of hypoPT symptoms (e.g., cramps, tingling, gastrointestinal symptoms) [15]. Studies have shown that a higher symptom burden is associated with lower QoL in hypoPT patients [41, 56]. Hillary et al. [38] reported that patients with hypoPT had significantly more symptoms using the HcSS compared to the control population. In Büttner et al. [14] patients with hypoPT had the highest symptom burden in *loss of vitality*, *pain and cramps*, and *numbness and tingling sensations* using the HPQ-28. The effect of current calcium levels and symptoms or QoL are controversially discussed within the literature. In the studies included in our systematic review 18 studies [5, 17, 19, 20, 26, 28, 29, 32, 33, 35–43] reported calcium levels of their study sample. While five studies [17, 19, 20, 40, 41] reported mean or median calcium levels within the reference range eleven studies [5, 26, 28, 29, 33, 35–37, 39, 42, 43] reported values below the reference range. Arlt et al. [32] stated that 18 of their 25 hypoPT had serum calcium levels within the accepted therapeutic range and in Hillary et al. [38] 43% of the participants stated that their calcium was in the normal range, while 44% had low or very low values and 3% had high values. An association between calcium levels and QoL was only observed in Hepsen et al. [37] with calcium levels being associated with *general health* and *vitality* of the SF-36. In the other included studies which investigated the association between calcium levels and QoL no association was found [5, 10, 19, 27, 32, 33, 35, 41, 43]. Addressing this, the European Society of Endocrinology clinical guideline [13] states that “Symptoms of hypocalcemia do not translate directly into serum calcium levels. Sudden fluctuations in serum calcium levels may cause symptoms, even if calcium levels are (almost) normal.”. HypoPT and its long-term conventional treatment might also lead to additional co-morbidities such as renal stones, cardiovascular disease, or calcifications, which might have a negative impact on QoL [3, 10]. Patients with hypoPT have reported that they have the feeling their disease is not understood [15, 65, 66] or that it is challenging to find an expert treating their disease [65]. This psychological burden might lead to a higher risk for anxiety and depression compared to norm populations [33, 65, 67–69], which might in turn influence QoL. In a study of 264 hypoPT patients, 16.3% of the patients reported that they had made use of at least one psychological service (e.g., psychologist, psychotherapist) due to their disease [65], with this share being the same or even higher compared to cancer patients [70, 71]. One aspect seldom assessed when the QoL of hypoPT patients is discussed is their ability of work. Büttner et al. [56] have shown that being unable to work due to the disease was associated with higher odds of reporting impairments of clinical importance for physical functioning, role functioning, and social functioning using the EORTC QLQ-C30. In the study by Astor et al. [5], it was stated that 40% of the hypoPT patients were receiving permanent or temporary social security benefits, while the proportion among the general Norwegian population is about 10% for permanent social security benefits and four percent for temporary benefits. In another study, 30% of the hypoPT who were working before their diagnosis stated the hypoPT had had an impact on their working situation ranging from reducing working hours to early retirement, or even being fired [65]. Among hypoPT patients, being hospitalized because of hypocalcemia is not uncommon. Proportions of hypoPT patients with at least one hospitalization because of hypocalcemia range from 5–44% between studies [65, 72–77]. Anaforoğlu et al. [72] found an association between hospitalizations or emergency department visits in hypoPT patients and QoL, making the fear or burden of hospitalization due to hypocalcemia another potential factor influencing QoL. As studies have shown an effect of hypothyroidism on QoL [78], one might question whether this was also present in the studies included in this systematic review and might explain some of the QoL impairments. Studies included in this systematic review could not find an association between thyroid stimulating hormone (TSH) levels and QoL in hypoPT patients [36, 42]. Additionally, the studies in postsurgical hypoPT populations had often matched controls who had also had total thyroidectomy, so that patients in both groups had to take medication for hypothyroidism [32, 34, 36, 41, 42]. Lastly, as thyroid cancer patients report impairments in QoL, the possibility exists that some of the impairments in postsurgical hypoPT patients might be attributable to the thyroid cancer itself and not hypoPT. Büttner et al. [34] and Mazoni et al. [41] only included thyroid cancer patients with and without hypoPT in their analysis and still found profound differences in QoL between hypoPT and non-hypoPT patients. While two studies [38, 42] stated that thyroid cancer might have an impact on QoL, other studies could not find an association between thyroid cancer and QoL in their hypoPT populations [36, 37, 56]. With longer disease duration it might be assumed that patients develop coping strategies or have accepted their disease resulting in fewer QoL impairments. Of the included studies six [26, 29, 33, 35, 38, 43] did not find an association between disease duration and QoL while three [14, 19, 37] studies found an association and the remaining studies did not include disease duration in their analysis. Hepsen et al. [37] and Tabacco et al. [19] found a correlation between disease duration and QoL in *vitality* and *mental health* respectively PCS using the SF-36. Using the disease specific HPQ-28 Büttner et al. [14] found a significant association between disease duration and various domains (*neurovegetative symptoms*, *loss of vitality*, *depression and anxiety*, *rapid heartbeat*, and *depressive symptoms*).

## Conclusion

Compared to norm populations or clinical controls, patients with hypoPT on standard treatment report impairments in QoL in various domains. The potential factors influencing QoL are most likely multifaceted and require regular monitoring as well as the inclusion of different professions.
